# Metastasis of renal cell carcinoma to the pancreas 11 years postnephrectomy

**DOI:** 10.11604/pamj.2018.30.53.15355

**Published:** 2018-05-23

**Authors:** Faten Limaiem, Saadia Bouraoui

**Affiliations:** 1 University of Tunis El Manar, Tunis Faculty of Medicine, 1007, Tunisia

**Keywords:** Pancreas, clear cell renal cell carcinoma, kidney, pathology

## Image in medicine

The pancreas is an unusual site for tumor metastasis, accounting for only 2 to 5% of all malignancies affecting the pancreas. A 67-year-old woman with a past medical history of right renal nephrectomy for renal cell carcinoma, eleven years ago, presented with nausea, diarrhea and vertigo. On admission, the patient was pale. Laboratory tests showed a low hemoglobin level (6,4 grams per deciliter). Abdominal ultrasonography revealed a well-defined, hypoechoic, homogeneous, vascular, lobulated mass in the tail of the pancreas. Computed tomography scan demonstrated a hypervascularized tumor of the pancreatic tail. The patient subsequently underwent a distal pancreatectomy with splenectomy. Grossly, the pancreatic mass was well-delineated and encapsulated measuring 9,5 x 6,5 cm and showed extensive hemorrhage (A). Histological examination of the surgical specimen revealed that the pattern of the tumour growth was predominantly solid, with formation of large nests and acini of tumor cells separated by a stroma that was endowed with prominent sinusoid-like vessels (B). The tumour cells were large ranging from optically clear, with sharply outlined boundaries, to eosinophilic tumour cells. The final pathological diagnosis was metastatic clear-cell renal carcinoma to the pancreas. Four peripancreatic lymph nodes were free of the tumor but the pancreatic resection margins were invaded by the tumour. Postoperative course was uneventful. Renal cell carcinoma metastasis should be considered in patients with a pancreatic mass as it gives the past history of renal cell carcinoma. Awareness of this entity and a high index of suspicion would help in proper diagnosis and treatment.

**Figure 1:**
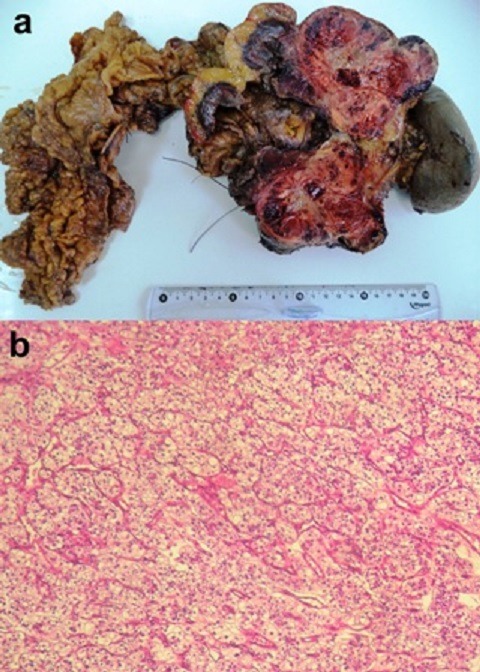
(A) gross appearance of metastatic clear renal cell carcinoma of the pancreas. On cut section, the pancreatic mass was well-delineated and variegated, measuring 9,5 x 6,5 cm and showed extensive hemorrhage; (B) tumour cells were arranged in solid nests and acini separated by a regular network of small thin-walled blood vessels (Hematoxylin and eosin, magnification x 100)

